# A 10-Year Social Media Analysis Exploring Hospital Online Support of Black Lives Matter and the Black Community

**DOI:** 10.1001/jamanetworkopen.2021.26714

**Published:** 2021-10-15

**Authors:** Yulin Hswen, Danyellé Thorpe Huerta, Circe Le-Compte, Jared B. Hawkins, John S. Brownstein

**Affiliations:** 1 Department of Epidemiology and Biostatistics, University of California, San Francisco; 2 Bakar Computational Health Sciences Institute, University of California, San Francisco; 3 Computational Epidemiology Lab, Harvard Medical School, Boston, Massachusetts; 4 Innovation Program, Boston Children’s Hospital, Boston, Massachusetts; 5 Department of Social Behavioral Sciences, Harvard T.H. Chan School of Public Health, Boston, Massachusetts

## Abstract

**Importance:**

Tensions around COVID-19 and systemic racism have raised the question: are hospitals advocating for equity for their Black patients? It is imperative for hospitals to be supportive of the Black community and acknowledge themselves as safe spaces, run by clinicians and staff who care about social justice issues that impact the health of the Black community; without the expression of support, Black patients may perceive hospitals as uncaring and unsafe, potentially delaying or avoiding treatment, which can result in serious complications and death for those with COVID-19.

**Objective:**

To explore how hospitals showed public-facing support for the Black community as measured through tweets about social equity or the Black Lives Matter (BLM) movement.

**Design, Setting, and Participants:**

Using a retrospective longitudinal cohort study design, tweets from the top 100 ranked hospitals were collected, starting with the most recent over a 10-year span, from May 3, 2009, to June 26, 2020. The date of the George Floyd killing, May 25, 2020, was investigated as a point of interest. Data were analyzed from June 11 to December 4, 2020.

**Main Outcomes and Measures:**

Tweets were manually identified based on 4 categories: BLM, associated with the BLM movement; Black support, expressed support for Black population within the hospital’s community; Black health, pertained to health concerns specific to and the creation of health care for the Black community; or social justice, associated with general social justice terms that were too general to label as Black. If a tweet did not contain any hashtags from these categories, it remained unlabeled.

**Results:**

A total of 281 850 tweets from 90 unique social media accounts were collected. Each handle returned at least 1279 tweets, with 85 handles (94.4%) returning at least 3000 tweets. Tweet publication dates ranged from 2009 to 2020. A total of 274 tweets (0.097%) from 67 handles (74.4%) used a hashtag to support the BLM movement. Among the tweets labeled BLM, the first tweet was published in 2018 and only 4 tweets (1.5%) predated the killing of George Floyd. A similar trend of low signal observed was detected for the other categories (Black support: 244 tweets [0.086%] from 42 handles [46.7%] starting in 2013; Black health: 28 tweets [0.0099%] from 15 handles [16.7%] starting in 2018; social justice: 40 tweets [0.014%] from 21 handles [23.3%] starting in 2015).

**Conclusions and Relevance:**

These findings reflect the low signal of tweets regarding the Black community and social justice in a generalized way across approximately 10 years of tweets for all the hospital handles within the data set. From 2009 to 2020, hospitals rarely engaged in issues pertaining to the Black community and if so, only within the last half of this time period. These later entrances into these discussions indicate that these discussions are relatively recent.

## Key Points

**Question:**

Do the top hospitals in the United States show public-facing support for the Black community on social media?

**Findings:**

In this cohort study including 281 850 tweets from 2009 to 2020, top hospitals in the United States did not communicate a high level of support for the Black community or topics related to social justice. Only 4 tweets related to the Black Lives Matter movement predated the killing of George Floyd.

**Meaning:**

These findings suggest that hospitals should consider how their social communication habits portray their commitment to a community.

## Teaser

This cohort study examines how and when US hospitals have expressed public-facing support for the Black community on social media.

## Introduction

Research has shown that patients who perceive their health care practitioners as concerned and compassionate experience better health outcomes.^1,2,3^ To that end, hospitals have increasingly leveraged social media to promote themselves as safe spaces for marginalized populations, such as Black individuals, to receive nondiscriminatory care.^3,4,5,6,7,8^ Our study seeks to examine whether hospitals express support for the Black population in relation to social justice issues on the social media platform Twitter.

This approach reflects the dominance of social media in addressing social justice issues online within the United States. In the US, a greater proportion of Black individuals use Twitter relative to White individuals, with the extensive conversations and communities of Black users and Black followers often referred to as *Black Twitter*.^8,9^ The hashtag synonymous with Black social justice issues, #BlackLivesMatter, and its diminutive #BLM, first appeared in 2012,^10^ and by 2016, it was the third most used social cause hashtag on the platform,^11^ appearing a mean of 17 002 times per day between 2013 and 2018.^12^ Although often used following fatal encounters with law enforcement, #BlackLivesMatter also became an important tool to raise awareness around health inequities in Black communities, such as HIV, adequate access to analgesia, and cancer screening.^13,14^

In 2020, #BlackLivesMatter took on new urgency owing to the convergence of several public heath crises that disproportionately impacted Black communities, including the COVID-19 pandemic and several high-profile cases of police violence against Black men and women. The Centers for Disease Control and Prevention estimates that cases of COVID-19 are 2.6-fold higher among the Black population than the White population,^15^ and that Black people with COVID-19 are 4.7-fold more likely to be hospitalized and 2.1-fold more likely to die than White people.^16,17^ Risk of death from COVID-19 is exacerbated in areas with larger populations of racial and ethnic minorities, such as Black individuals.^18^ This disparity reflects underlying social determinants of health: Black people are more likely to live in poverty, have limited access to health care,^19,20^ and work in essential, public-facing positions with limited social distancing and access to personal protective equipment.^21^ The ongoing incidents of police violence have resulted in demonstrations and discussions about the social and health impacts of systemic racism in America. While these events raised awareness, they also appeared to have heightened historic distrust of the medical community among underserved Black communities who expressed concerns about being exploited by researchers testing COVID-19 treatments and vaccines.^22,23,24^

It is imperative for hospitals to acknowledge themselves as safe spaces, run by clinicians and staff who care about social justice issues impacting the health of the Black community. Without the promotion of activism for the Black Lives Matter (BLM) movement, Black patients may perceive hospitals as uncaring and unsafe, possibly delaying or avoiding treatment,^25,26^ potentially resulting in serious complications and death for those with COVID-19.^27^ We explored how hospitals showed public-facing support for the Black community as measured through tweets about social equity and/or the BLM movement.

## Methods

This cohort study was approved by the Boston Children’s Hospital internal review board. Informed consent was waived because this study did not include any human participants.

Using the *Newsweek* world’s best hospitals 2020 list,^28^ we accessed each of the top 100 ranked hospitals’ official websites to retrieve their official account name on the social media platform. Since each account was obtained via hospital websites, it is reasonable to conclude that these hospital handles are run in an official capacity. The media relations department traditionally handles all outgoing communication for hospitals.

We pulled the most recent tweets from each handle starting from June 26, 2020, using the platform’s application programming interface until the interface would no longer return tweets for the handle. A total of 90 unique handles were collected from the top 100 hospitals. For hospitals that were part of the same health care system, the same handle represented several hospitals. One hospital did not have a handle linked to their website and was excluded from analysis.

To identify discussions related to the Black population, we reviewed the returned data to collect all hashtags used (including hashtags in retweeted or quoted tweets), conducting a keyword search for *Black*, *AfricanAmerican*, and *race*. Additionally, we conducted searches for specific hashtags related to BLM but not captured by the previous search (eg, *BLM* and *justice* to capture hashtags such as *racialjustice* or *justiceforGeorgeFloyd*).

Two of us (D.T.H. and Y.H.) manually reviewed all hashtags to remove any unrelated to the Black population (for example *blackbeans* would have been identified by our search because it has *black* in the hashtag but not included for our analysis). After a hashtag review, we pulled all tweets that included the identified hashtags to label if the tweet was BLM-related. To identify themes of analytic interest, data were coded by 2 of us (D.T.H. and Y.H.), and themes were built from codes.^29,30,31^ Four themes emerged through this thematic analysis^31,32^: (1) BLM, associated with the BLM movement; (2) Black support, expressed support for the Black population within the hospital’s community; (3) Black health, pertained to health concerns specific to and the creation of health care for the Black community; and (4) social justice, associated with general social justice terms that were too general to label as Black-focused (eg, “racism,” *peopleofcolor*, and *healthjustice*).

Both original tweets and retweets were extracted based on these groupings. We pulled all of the hashtags used in the identified tweets of interest, and based on these themes that emerged, we separated hashtags and manually categorized each tweet accordingly. Tweets that contained hashtags from more than 1 category were assigned to multiple categories. The 4 categories and tweet categorization were confirmed by all authors. We again manually reviewed a sample from each hashtag grouping to select the examples of tweets within each category that are provided in Table 1.

**Table 1.** Example of Tweets Labeled Within Each Category

Category	Examples of tweets
**Black Lives Matter**	At 1pm, faculty, staff and students will take a knee for 8 min and 46 s as part of the national #WhiteCoatsForBlackLives movement. [link]
	To demonstrate solidarity against racism, inequity and oppression, [institution] residents along with the larger [institution] community joined the #WhiteCoatsforBlackLives today to honor George Floyd and the other victims who have been killed as a result of police brutality. [link]
	We condemn the underlying culture of racism and violence in our country that has led us to where we are today. We’re so proud of all our people who participated in #WhiteCoatsForBlackLives, and we stand in solidarity with those who call for justice and reconciliation. [link]
**Black support**	At 17, [person] interviewed for the [institute] 6 y bachelor’s to MD program, declaring to then-dean [name], “if you give me the chance, I won’t let you down.” [name], the only African American & female in the class of 1935, graduated in the top 15. #BlackHistoryMonth [link]
	In 1944, Drs. Blalock & Taussig earned international acclaim for their new surgery, while Thomas went unnoticed. However, the success of the procedure could not have been accomplished without his research and operating room expertise. #BlackHistoryMonth [link]
	These black #physicians and #scientists are challenging, changing and writing history at [institution]. #BlackHistoryMonth [link]
**Black health**	Physicians & staff joined in a moment of silence [institution] as a commitment to improve the health & safety of people of color; show support for patients, colleagues, family, friends & their communities. #HealthCareForBlackLives [institution hashtags] [link]
	Our new study on #dementia may help to explain the higher incidence of dementia among #Hispanics and #AfricanAmericans [institution] [institution researcher] [link]
	Son of a slave and an indentured servant. Escaped to Canada on the #UndergroundRailroad. Graduated from the[institution] #medschool. Founded a medical school for #AfricanAmericans. Learn more about [physician name]: [link]
**Social justice**	Today, we joined #healthcare workers in [city] to raise awareness of #racialjustice and to show support for the Black Lives Matter movement. #doctorsforjustice 👊🏻👊🏼👊🏽👊🏾👊🏿 [link]
	A new op-ed published via @NYDailyNews, by CMO Dr [name] and vice chair of the Department of Medicine and gastroenterologist Dr [name], addresses how to ask better questions when having conversations about #race and #racism. [link]
	A recent statement from the @AmerAcadPeds says pediatricians are “deeply concerned about the effects of #racism on children.[”] So, how can you begin talking with children about #racialbias and injustice issues? [link]
**Uncategorized**	A patient with confirmed COVID19 is an inpatient at the [institution]. The patient is in stable condition.
	We are confident that we are using proper precautions with this patient. Learn more:[link]
	The coronavirus rapidly hijacks healthy cells in the respiratory tract and lungs. This can lead to pneumonia and acute respiratory distress syndrome (ARDS).
	See how coronavirus takes over the lungs.
	Learn more about COVID-19: [link] [link]
	[Institution hashtag]: What if your doctor’s stethoscope could predict your #heart’s future? Thanks to #ArtificialIntelligence, it’s already happening for some [institution] patients. [link]

Abbreviation: CMO, chief medical officer.

### Data Analysis

For each category, we explored 4 types of descriptive data. First, how many tweets contained the hashtags that defined the category? Second, how many handles were represented? Third, what were the tweet publication dates? And fourth, what were the most common hashtags?

Analyses were conducted in Python version 2.7.6 (Python Software Foundation). Data were analyzed from June 11 to December 4, 2020.

## Results

We collected a total of 281 850 tweets for 90 unique handles for the top 100 hospitals. Each hospital account returned at least 1279 tweets, with 85 handles (94.4%) returning at least 3000 tweets, and 74 handles (82.2%) returning at least 3200 tweets. The earliest tweet collected was published on May 3, 2009, and the latest was on June 26, 2020.

### Category Results

#### BLM

A total of 274 tweets (0.097%) used a hashtag to support the BLM movement, and a total of 67 handles (74.4%) had tweets in the BLM category. Of those 67 handles, 26 (38.8%) had only 1 tweet in this category. The first tweet was published on April 13, 2018 (5 years after the BLM movement and organization was founded). The date of the George Floyd killing, May 25, 2020, was investigated as a point of interest, and we observed that only 4 tweets that were labeled as BLM predated his killing. The median date of all tweets labeled within the BLM category was June 5, 2020 (IQR, 3 days). The most common hashtags used among these tweets included #GeorgeFloyd and 4 variations of #BlackLivesMatter, including 2 variations of #whitecoatsforBlacklives.

#### Black Support

We found 244 tweets (0.086%) with hashtags expressing acknowledgment or support of the Black community, and a total of 42 handles (46.7%) published tweets contained in the Black support category, 10 of which (23.8%) had only 1 tweet in this category. The first tweet was published on February 21, 2013, and the median tweet date was August 19, 2019 (IQR, 375 days). Popular hashtags recognized Black History Month and Black men and women in medicine.

#### Black Health

There were only 28 tweets (0.0099%), generated by 15 handles (16.7%), that used hashtags supporting Black community health concerns. Of these 15 handles, 13 (86.7%) had 1 tweet. The first tweet was published on February 12, 2018, and the median tweet date was December 17, 2019 (IQR, 475 days). Popular hashtags referenced African Americans, Black health, and Black lives.

#### Social Justice

We found 40 tweets (0.014%), produced by 21 handles (23.3%), containing hashtags in the social justice category. Of those 21 handles, 13 handles (61.9%) had 1 social justice tweet. The first tweet was published on April 10, 2015, and the median tweet date for the category was November 28, 2019 (IQR, 453 days). Popular hashtags referred to race and racism in general.

#### Uncategorized Tweets

Most tweets did not pertain to any of the categories listed (281 283 tweets [99.8%]). All hospital handles were represented in this group. The first tweet was published on May 3, 2009, and the median tweet date was December 18, 2018 (IQR, 812 days).

### Summary Results

**Figure 1.**
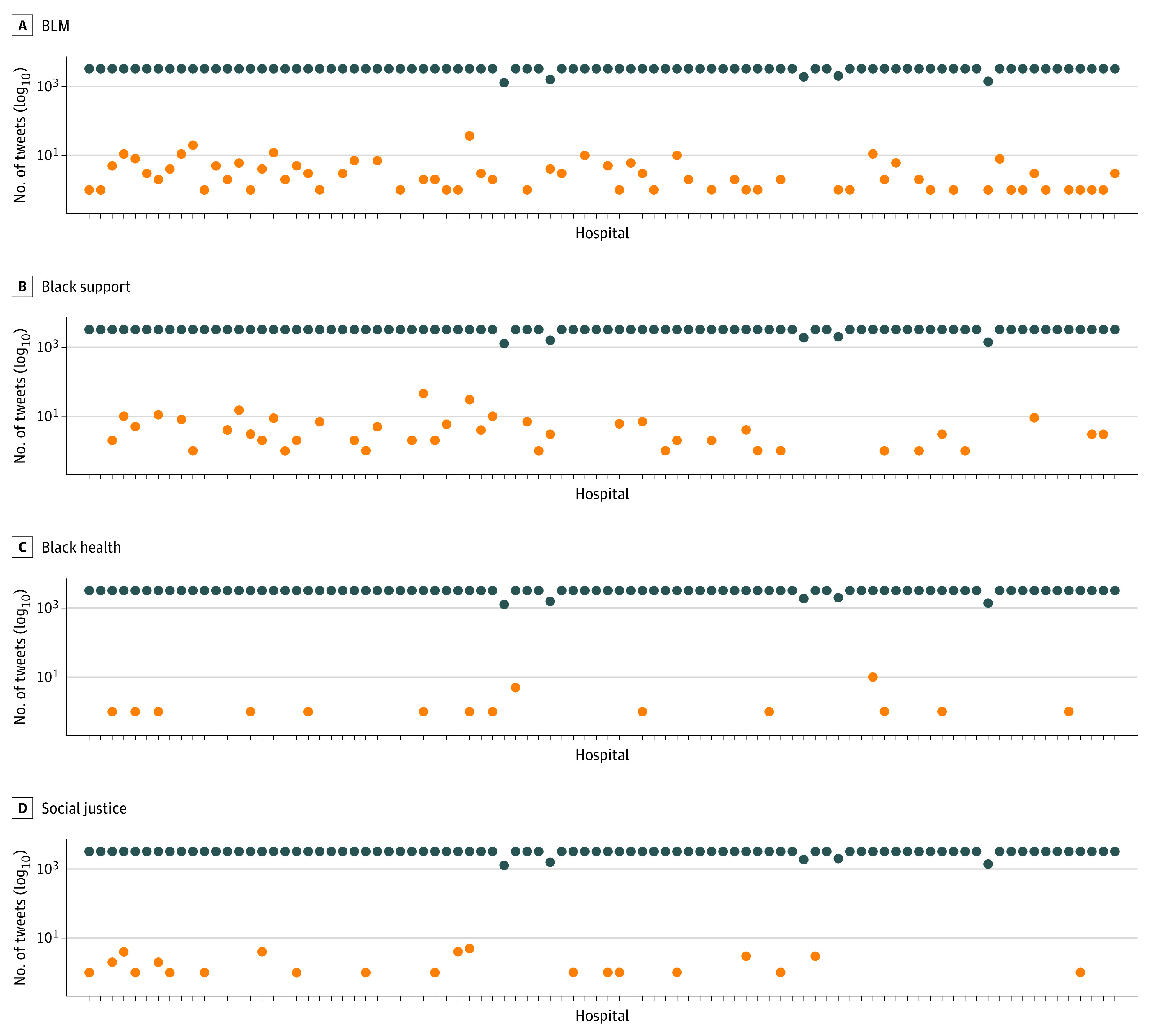
Tweets Per Hospital Per Category Total amount of tweets per hospital (log_10_) in each category (orange dots) and uncategorized tweets (blue dots). The uncategorized hospitals are ordered in the assigned *Newsweek* ranking^28^ from left to right. Hospitals without an orange dot did not publish any tweets labeled within that category.

**Figure 2.**
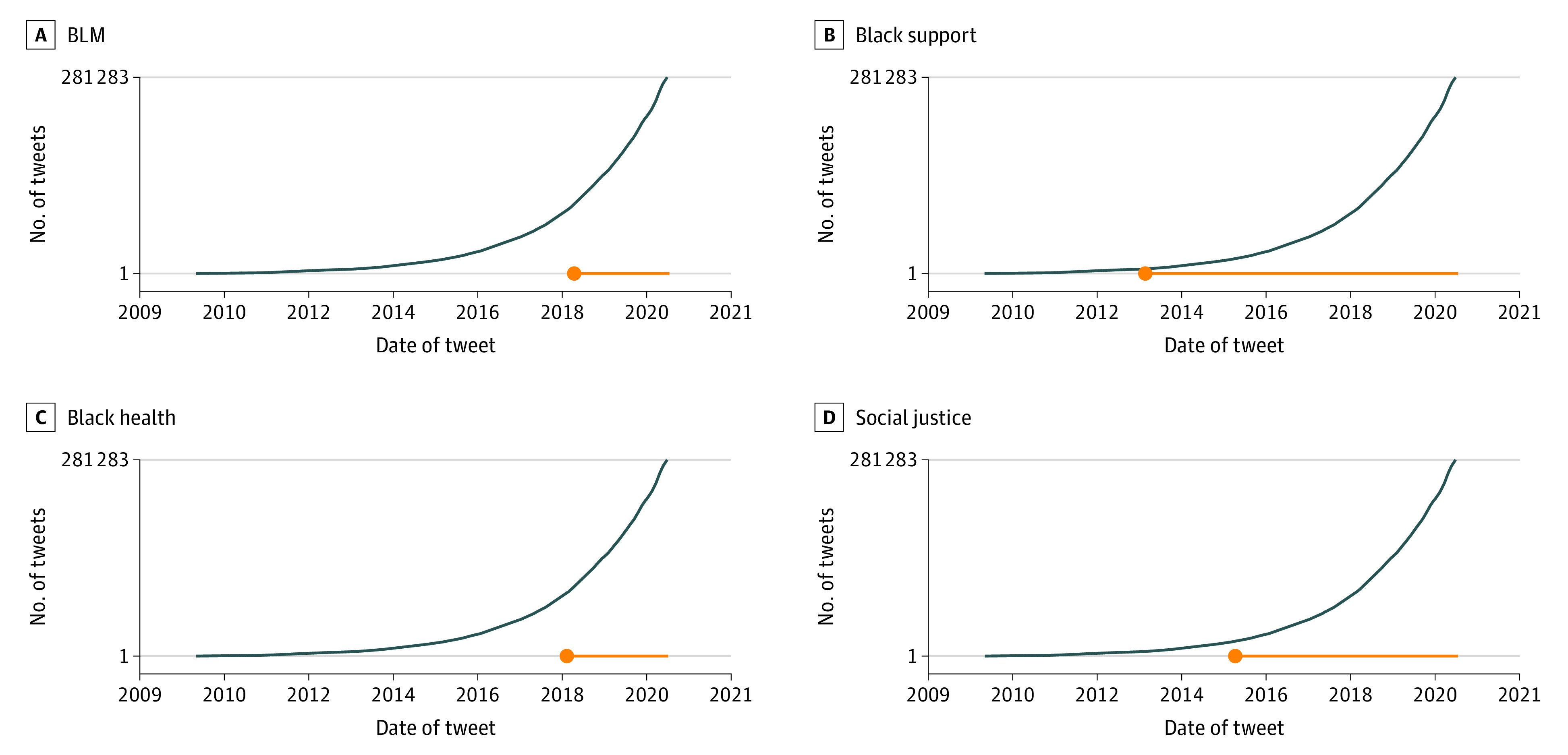
Cumulative Number of Tweets Across Time Cumulative number of tweets across all hospitals over time for each category (orange line) and uncategorized tweets (blue line). The timeline ranges from May 3, 2009 (first collected tweet), to June 26, 2020 (last collected tweet). Dots indicate the earliest publication date of tweets within that category.

Results for each category of tweets are summarized in Table 2. Figure 1 shows the number of tweets for each hospital handle for each category. For added context, we plotted each handle’s total number of uncategorized tweets on each category’s plot. The labeled tweets per hospital ranged from 0 to 46 tweets, and the median date for each category was in 2019, within 1 year of when the data was collected. The uncategorized tweets per hospital ranged from 1279 to 3250 tweets, and the median date was in 2018. Figure 2 shows the cumulative number of tweets across time from May 3, 2009, to June 26, 2020. Again, we added uncategorized tweets to each category’s plot for added context.

**Table 2.** Numbers of Tweets by Categories and Top 5 Hashtags

Category	Tweets	Hospital handles, No. (%)	Date of first tweet	Median date (IQRt, d)	5 Most common hashtags used (No. times used)
Black Lives Matter	274	67 (74.4)	4/13/18	6/5/20 (3)	#whitecoatsforBlacklives (242) #BlackLivesMatter (125) #whitecoats4Blacklives (21) #BLM (16) #GeorgeFloyd (16)
Black Support	244	42 (46.7)	2/21/13	08/19/19 (375)	#BlackHistoryMonth (179) #Blackmeninmedicine (70) [an institution hashtag] (31) #diversity (16) #Blackwomeninmedicine (14)
Black Health	28	15 (16.7)	2/12/18	12/17/19 (475)	#AfricanAmerican or #AfricanAmericans (15) #healthcareforBlacklives (10) #Blackhealth (3) #Massachusetts (2)
Social Justice	40	21 (23.3)	4/10/15	11/28/2019 (453)	#racism (24) #race (9) #publichealth (4) #COVID19 (4) #whitecoatsforBlacklives (4)
Uncategorized	281 283	90 (100)	5/3/09	12/18/18 (812)	#COVID19 (11 660) #cancer (4404) #coronavirus (3072) #breastcancer (2251) #health (1896)

## Discussion

The findings of this cohort study reflect the low signal of tweets regarding the Black community and generalized social justice from hospitals in the US. It is evident from the lack of signal for labeled tweets relative to uncategorized tweets. This lack of discussion surrounding the Black community is a trend across approximately 10 years of tweets across all hospitals within our data set. Of the small percentage of tweets that had Black community or social justice labels, the earliest tweets are at least 6 years later than the earliest tweet published in the data set. This, along with the median dates within a year of collection, indicate that these discussions are relatively recent.

Most hospitals and hospital systems focus their tweets on somatic health. While some hospitals framed COVID-19 as a public health crisis and noted its disparate impact in Black communities, the signal was low. Hospitals tended to separate messages about Black health and COVID-19. Although we cannot determine the intention with these tweets, the hospitals’ separation of BLM and COVID-19 effectively elides the disparate impact of COVID-19 in Black communities.

Only 4 tweets involving BLM occurred prior to the killing of George Floyd on May 25, 2020, while 270 tweets occurred after. Hospital participation in the national response to his killing is evidenced by the most popular hashtag in the group (#GeorgeFloyd). It is not possible to determine whether the increases in tweets related to BLM will continue at these hospitals or reflect a response to a specific event.^33^ Institutions may consider ongoing use of social justice hashtags to be outside their scope or overly political, but hospitals can powerfully leverage social justice to demonstrate concern for the health and well-being of the Black members of the communities they serve.^33^ Whether intentional or not, these top hospitals are not communicating nor demonstrating outwardly that they care about issues and concerns specific to the Black community. Because of this, Black communities may not feel represented at top hospitals. Erasure^34^ from public discourse has been shown to be negatively associated with the quality of care delivered to marginalized populations and with the uptake of care by these populations.^35^ A 2021 study by Kiang and Tsai^36^ examined the public communication statements of 56 leading US medical schools after the George Floyd killing. Kiang and Tsai found that most medical schools failed to make reference to the killing of George Floyd by the police and historical racism against Black people.^36^

Social justice has been shown to be a powerful tool in addressing health inequities in Black communities, motivating changes in policy to improve public health outcomes^37,38,39^ related to access to care,^40,41^ quality of care delivery,^42^ and overall health care outcomes.^43^ In addition, hospitals that leverage social justice messaging and hashtags may be able to further the reach of their tweets and better engage communities. Indeed, some studies, such as a 2018 study by Edrington,^44^ have suggested that tweets that contain calls to action related to social and health policy are more likely to be engaged and retweeted.

We recognize that our analysis is based only on hashtags and not on text, creating conservative grouping. However, a critical consideration is that general social media etiquette includes hashtags, especially when a user wants to ensure that their message is clearly stated and grouped with messages like it for increased visibility. Hospitals tend to employ professional communications teams versed in social media norms and likely are well versed in labeling messages for public consumption. While conservative, we believe these tweets capture the social justice messages that hospitals wanted amplified via signal boosting through hashtags.

To our knowledge, this is the first study that has looked at hospital advocacy of Black social justice issues and BLM in particular. Additionally, this study illustrates a baseline of how hospitals are tweeting. While there were few communications about BLM in this set of tweets, our study could provide guidance for further work concerning how online BLM advocacy may be associated with trust or improved health outcomes in the Black population a hospital serves. By providing hypotheses for future studies, our work may advance research to define the importance of trust and how it relates to promotion of Black community and social justice issues. It also may encourage hospitals to expand their social justice advocacy of historically underserved populations in an effort to increase community trust and improve the health outcomes of affected populations.

Further analysis needs to be conducted to find other potential signals and avenues that communicate effective engagement (eg, examining other social media sites or community-based outreach). We hope that our work inspires future research on how a hospital’s advocacy and outward-facing discussion centered on health disparities and social injustices could help to advocate for and support the Black population in terms of trust and safety. We hope that future analyses contextualize our findings further by exploring whether community- or minority-based hospitals are more likely to engage in social justice, whether there are differences in levels of hospital engagement by minority group, and sentiment and linguistic properties that extend beyond hashtags. Eventually, it is our aim that this analysis will be expanded to include more US hospitals varying in conventional ranking and community demographics served.

### Limitations

This study has some limitations. It is possible that we would have seen more hospitals engaged in these topics if we had expanded our method to have less conservative grouping. We also may not have included all hashtags associated with BLM (eg, #protest, #policebrutality). Again, we argue that if a hospital wanted to publicly engage in these topics, they would have used hashtags central to movements to take advantage of signal boosting.^45,46^

We also recognize that we have included only the top 100 ranked hospitals according to *Newsweek*; thus, our results may not be generalizable to all hospitals in the US. We did not include community- or minority-based hospitals as a comparison to explore if these hospitals that do not rank high in conventional US hospital rankings would rank high in terms of advocacy.

## Conclusions

The findings of this cohort study help to shed light on how hospitals use social media and hashtags to address social justice issues impacting the Black communities they serve. It is important for health organizations to realize how they engage with their communities via social media. These brief engagements can communicate an institution’s dedication to the health and well-being of the populations they serve. Such engagements are particularly important among underserved and marginalized groups, which historically have not trusted mainstream health organizations and are, in general, heavy users of communications platforms. Health organizations that thoughtfully address social justice issues online may be able to build trust and bolster uptake of their services among these groups and help to improve their health outcomes overall.
